# Using Virtual Reality to Complement Paper‐and‐Pencil Tests to Assess Visual Unilateral Spatial Neglect: A Feasibility Study

**DOI:** 10.1155/bn/6684846

**Published:** 2026-06-08

**Authors:** Wieteke Sauter, Peter van der Wurff, Nikita van Schijndel, Pim Abbink, Noël Keijsers, Juha Hijmans, Kenneth Meijer, Anke van Bladel, Maarten R. Prins

**Affiliations:** ^1^ Research and Development, Military Rehabilitation Center, Aardenburg, the Netherlands; ^2^ Rehabilitation Center Klimmendaal, Arnhem, the Netherlands; ^3^ Rehabilitation Center Revant, Breda, the Netherlands; ^4^ Dept. of Research, Sint Maartenskliniek, Nijmegen, the Netherlands, maartenskliniek.nl; ^5^ Dept. of Rehabilitation and Dept. of Sensorimotor Neuroscience, Donders Institute for Brain, Cognition and Behaviour, Radboud University, Nijmegen, the Netherlands, ru.nl; ^6^ Dept. of Rehabilitation Medicine, University Medical Center Groningen, Groningen, the Netherlands, umcg.nl; ^7^ Dept. of Nutrition and Movement Sciences, Maastricht University Medical Centre+, Maastricht, the Netherlands, mumc.nl; ^8^ Dept. of Rehabilitation Sciences, Ghent University, Ghent, Belgium, ugent.be; ^9^ Dept. of Rehabilitation Sciences, KU Leuven–Brugge, Bruges, Belgium

**Keywords:** extrapersonal space, neuropsychological assessment, stroke, unilateral spatial neglect, virtual reality, visual search

## Abstract

**Background:**

Visual neglect is one of the most frequent neuropsychological consequences of acute brain damage. Generally, paper‐and‐pencil tests, administered in the peripersonal space, are used to establish the presence of neglect. However, the multidimensional nature of neglect makes the diagnostic process challenging. Neglect varies, among other things, in spatial regions; that is, personal, peripersonal, and extrapersonal space.

**Objective:**

In this feasibility study, we aim to establish the reliability and validity of a newly developed Functional ExtraPersonal Space Neglect Test (FEPSNeT) executed on a virtual reality system.

**Methods:**

Stroke patients with a first stroke in the right hemisphere, treated in four rehabilitation centers in the Netherlands equipped with a GRAIL (Gait Real‐Time Analysis Interactive Lab), were included in this study. The participants performed the FEPSNeT twice a week on separate days. The Line Bisection Test (LBT), the Star Cancelation Test (SCT), and the Catherine Bergego Scale (CBS) were also administered.

**Results:**

The test–retest reliability of the FEPSNeT was nearly acceptable. The construct validity of the FEPSNeT was considered sufficient because the subjects with the most asymmetrical reaction times all responded slower to stimuli on their left side. There was no significant correlation between the FEPSNeT and paper‐and‐pencil tests (LBT and SCT), and there was a low but significant correlation between the FEPSNeT and CBS.

**Conclusions:**

The FEPSNeT appears to assess neglect in a different region (extrapersonal space) than paper‐and‐pencil tests (peripersonal space), and therefore, it can be of added value to paper‐and‐pencil tests in the assessment of visual neglect.

## 1. Introduction

Visual unilateral spatial neglect (VUSN), henceforth referred to as *neglect*, is one of the most frequent neuropsychological consequences of acute brain damage [1]. Approximately 50% of right‐sided stroke patients and 30% of left‐sided stroke patients demonstrate symptoms of neglect [2, 3]. These patients show decreased attention to events and stimuli at the contralesional side, resulting in slower or even absent responses [2, 3]. Neglect has been associated with slower motor recovery [4, 5] and disability in activities of daily living (ADL) [4–6]. Patients with neglect are more dependent in self‐care, transfers, and locomotion, particularly in the subacute phase after stroke. This results in extended admission times in hospitals and rehabilitation centers [5] and a higher dependency on caregivers after discharge [7]. Since early treatment of neglect can be effective, an accurate and fast diagnosis of neglect is important to adjust the rehabilitation treatment accordingly [7].

The multidimensional nature of neglect makes the diagnostic process challenging. Neglect varies (among other things) in regions of space; that is, personal, peripersonal, and extrapersonal neglect [8, 9]. Personal neglect is neglect for one′s own body, peripersonal neglect is neglect for events and stimuli within reaching distance, and extrapersonal neglect is neglect for events and stimuli relatively far from the observer [10]. It has been found that there can be a considerable difference in neglect severity between these regions within a patient [11, 12].

Generally, the presence of neglect is established using neuropsychological tests. However, nurses or caregivers often observe neglect during the ADL in patients where neglect was not identified by these tests [13]. While paper‐and‐pencil tests are often designed to establish neglect in the peripersonal space, the Catherine Bergego Scale (CBS) is aimed at capturing the three dimensions of space in a questionnaire that covers signs of neglect during 10 ADL tasks. Although the CBS is a reliable and valid instrument to assess behavioral neglect in stroke patients [14], a disadvantage can be that it is time‐consuming to observe and score all 10 ADL items [15]. As a result, it regularly occurs that some items are not completed because they have not yet been observed within the time frame in which the test is administered [16]. Another disadvantage is the subjectivity of the CBS; the test does not clearly define the observational context for each item, which may lead to considerable variation in how clinicians administer the assessment [17]. This disadvantage could, however, be addressed by using the KF‐NAP to administer the CBS. The structured guidelines provided by the KF‐NAP help ensure a more consistent observational context across evaluators, thereby enhancing the standardization and reliability of the assessment process [17].

An additional assessment for extrapersonal hemispatial neglect should be considered in the diagnosis of neglect [13, 15, 18]. Therefore, we developed a Functional ExtraPersonal Space Neglect Test (FEPSNeT) in which patients should respond to appearing targets in a virtual environment as fast as possible. During the test, reaction times to stimuli in the extrapersonal space at both the lesional and contralesional side were determined. In this study, we aim to establish the reliability and validity of this test. Test–retest reliability was determined. The construct validity of the FEPSNeT was evaluated by determining if response times to stimuli were related to the side of the lesion. We expected that patients with a right‐sided lesion would tend to respond slower to stimuli presented on the left side of the screen. The concurrent validity was assessed by comparing the FEPSNeT with commonly used neglect tests, that is, paper‐and‐pencil tests and the CBS. We expected the correlation between the FEPSNeT and paper‐and‐pencil tests to be lower than the correlation between the FEPSNeT and CBS, because paper‐and‐pencil tests only evaluate neglect in the peripersonal space, whereas the CBS evaluates neglect in the personal, peripersonal, and extrapersonal space, which would suggest some overlap with the FEPSNeT.

## 2. Methods

The medical ethical committee of UMCG (University Medical Center Groningen) determined that no medical ethical judgment was required for this study because data were collected during usual care (METc2016/306 UMCG). The ethical standards of the Helsinki Declaration of 1975 and revised in 2014 were followed (General Assembly of the World Medical Association, 2014All), hence, all subjects participated voluntarily in the study and provided informed consent. This cross‐sectional study conforms to all STROBE guidelines and reports the required information accordingly [19].

### 2.1. Participants

Stroke patients included in this multicenter study were treated in four rehabilitation centers in the Netherlands equipped with a GRAIL (Gait Real‐Time Analysis Interactive Lab): Klimmendaal Arnhem, Revant Breda, Sint Maartenskliniek Nijmegen, and Military Rehabilitation Center Aardenburg Doorn, from July 2018 to February 2020. Inclusion criteria were first stroke in the right hemisphere, age older than 18 years, and a Functional Ambulation Categories (FAC) score of 3 or above. Exclusion criteria were severe cognitive and/or communicative impairments, visual impairments (e.g., hemianopsia and dementia) and other neurological problems (e.g., Parkinson′s disease). Note that patients were not selected on symptoms of neglect. To eliminate selection bias, all subjects with right hemisphere damage were approached in the centers to participate in this study.

### 2.2. Procedure

The study protocol covered 1 week and consisted of two sessions. Prior to the first session, demographic and stroke characteristics were collected, and the CBS was administered by the nursing staff. The nurses received instructions from the first author prior to the start of the study to ensure they were able to administer the CBS appropriately. In the first session, the Line Bisection Test (LBT), Star Cancellation Test (SCT), and FEPSNeT were performed. In the second session, the FEPSNeT was performed again (Table 1). Each center has one or two operators who have expertise in treating neurology patients using the virtual system. They administered the FEPSNeT, the LBT, and the SCT.

**Table 1:** Overview of outcome measurements in this study.

	S0	S1	S2
**Demographic and stroke characteristics**	X		
*Age*, *gender*, *type of stroke*, and *time poststroke*			
**Behavioral assessment**	X		
*Catherine Bergego Scale*			
**Paper-and-pencil tests**		X	
*Star Cancellation Test and Line Bisection Test*			
**Virtual reality assessment**		X	X
*Functional ExtraPersonal Space Neglect Test*			

### 2.3. Outcome Measures

#### 2.3.1. Demographic and Stroke Characteristics

In the first week of the study, demographic information and stroke characteristics were collected from the patient′s medical record, that is, age, gender, type of stroke (ischemic or hemorrhagic), and time poststroke.

#### 2.3.2. Assessment of Neglect

Participants were screened for neglect with two paper‐and‐pencil tests, the SCT and the LBT, and with the CBS. The paper‐and‐pencil tests were completed by the participants during the first session on an 8.5^″^ × 11^″^ piece of paper placed right in front of the patient. The CBS was administered during the test week by the nursing staff.

The SCT contains 54 small stars and 75 distractors (52 large stars, 13 letters, and 10 short words). Patients were instructed to cross out all small stars. A *laterality index* was calculated by dividing the number of small stars crossed out on the left side of the paper by the total number of crossed out small stars. Scores ≤ 0.46 implied VUSN to the left side, and scores ≥ 0.54 implied VUSN to the right side [20]. There was no time limit to fill out the SCT.

During the LBT, participants were instructed to divide three lines with a length of 182 mm into two equal parts using a vertical stripe. The test was scored by calculating the deviation from the true center of the line. The maximum score for each line was three, and the total score was nine points. This maximum score was reached if the patient′s mark in all three cases lies within 12.75 mm to the left or right of the center. Deviations from the center were scored by reference to deviation scores on the scoring templates used in the behavioral inattention test, and the cutoff point that indicated neglect was 7 or fewer points [21]. As for the SCT, there was no time limit to complete the LBT.

The CBS assesses neglect during everyday life situations. Ten functional tasks (e.g., dressing, eating, and finding the way) were observed and scored by the nursing staff of each center. The maximum score of the test was 30 points, three per item and with a score of six or higher as the threshold value for neglect [22]. Unscored items were excluded from the total score. The total score was calculated by summing the item scores, dividing this sum by the total number of scored items, and multiplying the result by 10. Because this scale is scored manually by the nursing staff, observer bias may play a role. However, the observers are not aware of the research aims or hypotheses of this study.

#### 2.3.3. FEPSNeT

**Figure 1:**
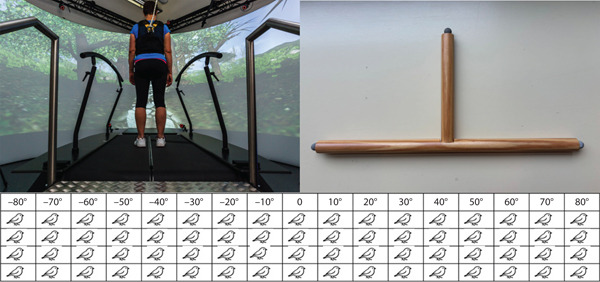
Functional ExtraPersonal Space Neglect Test (FEPSNeT) on the GRAIL. In the upper left corner, the GRAIL setup in the Military Rehabilitation Center, Aardenburg. The GRAIL is a virtual reality system consisting of a treadmill, a 180° round screen on which a virtual environment is projected, and a motion capture system. In the upper right corner, the wooden tool is used to control the FEPSNeT. On the three ends of this wooden tool, reflective markers are placed. Because these markers are seen by the motion capture cameras, a ball can be controlled by pointing the middle of the tool toward the desired position on the screen. With this ball, the appearing birds must be hit as quickly as possible. In addition, at the bottom, the schematic overview of the FEPSNeT. The screen is divided into 68 cells, and in each cell, a bird will appear once.

The FEPSNeT is a newly developed test on the GRAIL, created by the authors of this paper (M.R.P. and W.S.). The GRAIL is a virtual reality system that consists of a treadmill, a 180° round screen and a motion capture system (Figure 1—upper left corner). The distance between the screen and the participant is about 2.5 m. During the FEPSNeT test, a forest path was projected with an optic flow of 3 km/h, as if the subject was sliding straight ahead along the forest path. Moreover, although the GRAIL is equipped with a treadmill, it was not used. The subject on the GRAIL could move a virtual yellow ball projected on the screen using a wooden tool by pointing the middle of the tool toward the desired position on the screen (Figure 1—upper right corner). The aim of the test was to hit birds that appeared one by one on the screen with this ball as quickly as possible. Once appeared, a bird would remain on the screen until hit or until it had been visible for 60 s without being hit. The screen was divided into 17 vertical columns (10° per column, with the middle column straight in front of the subject) and four horizontal rows, resulting in a total of 68 cells. During the test, a bird would appear in each cell in a random order (Figure 1—bottom). Each consecutive bird would appear 0.1–3 s after the previous bird was either hit or missed after 60 s. The response time per cell was recorded. From the response time per cell, an average response time to the birds appearing on the left and right parts of the screen was calculated. The response times to the birds in the middle column were discarded from further analyses.

Using the reaction times, the space asymmetry index for time (SAI_Time_) as introduced in the study of Borsotti et al. was calculated. This SAI_Time_ is a ratio of the reaction times toward the left and right spaces, weighed for the patient′s overall capacity. Values approaching −1 would indicate neglect of the left extrapersonal space (longer reaction times to the left side), and values approaching +1 would indicate neglect of the right extrapersonal space (longer reaction times to the right side) [15].

SAITime=RT mean right−RT mean leftRT mean left+RT mean right

The SAI_Time_ was calculated by the system and clinicians have the option to project it directly onto the screen, which is an advantage. This means that the test result is immediately visible. However, during the study, we opted not to do so in order to avoid influencing the research data. Because the test was administered and assessed completely automatically, the observer bias did not occur. Because we had no golden standard for neglect in extrapersonal space, we had to select an arbitrary SAI cutoff for neglect on the FEPSNeT. The presence of neglect was not a selection criteria in this study, and therefore, we expected the SAI of the majority of the subjects would be clustered around zero. We aim to select two cutoff points with an equal distance to a perfectly straight SAI (e.g. −0.1 and 0.1 or −0.3 and 0.3). Subjects with scores outside this range would be labeled as subjects with neglect in the extrapersonal space (to the left and right side, respectively). To determine the cutoff point, we aim to (1) primarily label subjects with a negative SAI, indicating neglect on the left side (since we exclusively included patients with lesions in the right hemisphere) while (2) trying to keep a margin of error around the subjects not labeled with neglect.

### 2.4. Statistical Analyses

To analyze the test–retest reliability between the FEPSNeT scores of sessions one and two, ICC estimates and their 95% confidence intervals were calculated based on a single‐rating, absolute‐agreement, two‐way mixed‐effects model [23]. An ICC of greater than 0.7 was deemed acceptable.

For the construct validity a histogram with the SAI of each subject was plotted to assess whether the data distribution (SAI) was skewed to the left, which would indicate that some participants would demonstrate relatively long reaction times and hence neglect on the left side.

To assess the concurrent validity, Spearman′s rank correlation coefficients were computed between the paper‐and‐pencil tests, the CBS tests, and the FEPSNeT.

For all statistical analysis, we used SPSS Version 27 (Armonk, New York: IBM Corp, United States), and the level of significance was set at *p* < 0.05. Moreover, if data were missing, for example when subjects dropped out during the measurement week, the subject was excluded from the dataset.

## 3. Results

### 3.1. Demographic and Stroke Characteristics

Forty‐three stroke patients, both ischemic (*N* = 31) and hemorrhagic (*N* = 12), were included in this study. The sample consisted of 33 males and 10 females with a mean age of 62 years (SD 10.4, range 36–79) (Table 2). None of the participants reported other neurological or vision problems. One subject dropped out due to discontinuation of rehabilitation, and there was no missing data. The median (IQR) time to complete the test in minutes was 3.43 (2.57–5.46).

**Table 2:** Demographic and stroke characteristics (*N* = 43).

Outcome	*N*(%)	Mean	SD
**Age (years)**		62	10.4
**Gender**			
Male	33 (77%)		
Female	10 (23%)		
**Time poststroke onset (days)**		45	19
**Stroke type**			
Ischemic	31 (72%)		
Hemorrhagic	12 (28%)		

### 3.2. Test–Retest Reliability

The ICC(3,1) of the FEPSNeT was 0.70 (95% CI: 0.50–0.83), indicating a nearly acceptable reliability.

### 3.3. Construct Validity

**Figure 2:**
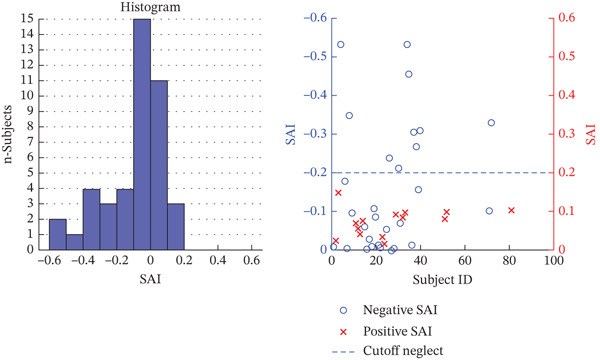
Distribution of space asymmetry index (SAI). In the left corner, a histogram. In each column, the number of subjects who fall within the range of the determined space asymmetry index value in 0.1 scale is shown. Most SAI were concentrated around zero, with 10 subjects scoring below an SAI of −0.2. In the right corner, a scatterplot. Subjects with a negative SAI score are indicated with a blue circle (left *y*‐axis), positive SAI scores are indicated with a red cross (right *y*‐axis). The cutoff point for neglect was set on SAI ±0.2. In this plot, the 10 outliers above this line were all negative values, indicating a neglect on the left side.

The median (IQR) of the absolute reaction times (maximum 60 s) was 3.28 s (2.61–5.09). There was a significant negative correlation between SAI and mean reaction time; *r* (42) = −0.497, *p* < 0.001. The histogram in Figure 2 (left corner) shows that, as expected, SAI was skewed to the left with most scores concentrated around a SAI of zero. In Figure 2 (right corner), a scatter plot is shown with the SAI of all subjects. As already described, we aim to primarily select subjects with a negative SAI while trying to stay outside the range of the majority of the participants. Therefore, we chose a SAI cutoff point for neglect of +/−0.2, resulting in 10 subjects with neglect on the left side in the extrapersonal space based on the FEPSNeT.

### 3.4. Concurrent Validity

**Figure 3:**
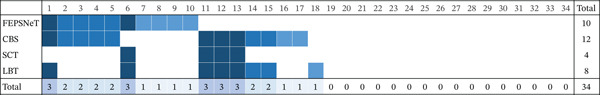
Results of the different neglect tests. In this figure, it is accentuated per subject whether the score on the neglect test was positive.

Figure 3 shows that most subjects (*N* = 25, 58%) scored negative for neglect on all the tests, indicating that they would not be diagnosed with neglect based on the usual care neglect tests. Conversely, none of the subjects scored positive on all the neglect tests. Of all subjects with some indication of neglect (*N* = 18), 67% scored positive for neglect on more than one test. However, the combination of positive tests varied greatly between subjects. Particularly, the combination of a positive FEPSNeT and paper‐and‐pencil test was rare. Only 11% (*N* = 2) of subjects with some form of neglect scored this combination.

The FEPSNeT correlated significantly with the CBS with a Spearman rank order correlation of 0.370, *p* = 0.015, but not with the paper‐and‐pencil tests. The CBS also correlated significantly with the LBT (*r* = 0.419, *p* = 0.005), but not with the SCT. The paper‐and‐pencil tests significantly intercorrelated (*r* = 0.67, *p* < 0.001) (Table 3).

**Table 3:** Spearman rho for concurrent validity.

	Star Cancellation Test	Line Bisection Task	Catherine Bergego Scale	FEPSNeT SAI Session 1
Star Cancellation Test	1	—	—	—
Line Bisection Task	0.670 *p* < 0.001	1	—	—
Catherine Bergego Scale	0.281 *p* = 0.068	0.419 *p* = 0.005	1	—
FEPSNeT SAI Session 1	0.013 *p* = 0.933	0.020 *p* = 0.900	0.370 *p* = 0.015	1

## 4. Discussion

In this study, we aim to establish test–retest reliability, construct validity and concurrent validity of the FEPSNeT. The test–retest reliability was nearly acceptable, suggesting sufficient reliability to use the test in clinical practice. In addition, this was in line with the test–retest reliability of the SCT and the LBT (respectively, ICC = 0.89 and ICC = 0.97) [24]. The FEPSNeT appeared to measure the construct neglect adequately, because the subjects with the largest asymmetries in reaction times responded slower to stimuli on the left side, which was to be expected given that we included right‐sided stroke patients. There was no significant correlation between the FEPSNeT and paper‐and‐pencil tests (SCT and LBT), and there was a low but significant correlation between the FEPSNeT and CBS. This difference in correlations supports our hypothesis that the FEPSNeT evaluates neglect in a different region (i.e., extrapersonal space) than the paper‐and‐pencil tests (i.e., peripersonal space) and that the CBS evaluates a combination of regions. This is further supported by the observation that the FEPSNeT did not detect neglect in some cases in which other measures indicated its presence, and vice versa. Consequently, combining the FEPSNeT with traditional paper‐and‐pencil tasks and the CBS appears to enhance diagnostic sensitivity, as these assessments may detect complementary aspects of neglect. Not only the assessed regions of neglect differ between these tests, but also the consideration of reaction times. For example, when administering paper‐and‐pencil tests, the time it takes to complete these tests is not included in the score. This implies that patients who need more time to cross out stars on the left side of the paper during the Star Cancellation Test will not be diagnosed with neglect if they succeed in the task. In the FEPSNeT, the participants must hit the appearing targets as fast as possible. The outcome measure of the FEPSNeT is the reaction time, adding time pressure. On the one hand, this could contribute to the fact that no significant correlation was detected between the paper‐and‐pencil tests and the FEPSNeT. However, we know from previous research that computerized tests, in which reaction times and accuracy are measured, have advantages over traditional paper‐and‐pencil tests [25]. On the other hand, including reaction times and dynamic targets in neglect tests could be of added value, because this reflect daily life situations where patients need to act in a dynamic environment in which tasks are constantly performed under time pressure.

Recently new tests to assess neglect have been described in literature. One example is the Mobility Assessment Course (MAC) [3], during which participants have to perform a simple wayfinding task (walking or riding in the wheelchair) in a corridor while finding targets and reporting them. The advantage of this test is that it is performed in a corridor that is used by other people at the time of assessment. This a real‐world setting, including “distractors” that force you to divide your attention. However, this also implies that the test is less standardized. Depending on how busy the corridor is, there may be variation between two test moments, and subsequently, the type of corridor is also different everywhere, making it impossible to compare scores between departments and locations. In contrast, the FEPSNeT is highly standardized because it is executed in a virtual reality environment that simulates the “real world” in the same manner each time the FEPSNeT is performed. Virtual reality also allows to add similar distractors and multitasking as in the MAC, such as adding passing pedestrians to the forest path and/or perform the test while walking [18]. In the future, dual tasks or distractors could be added to the FEPSNeT, to ensure the cognitive load increased, and there is less room for compensation strategies.

There are some limitations in this study, for example, we did not measure a group of healthy participants. This would have enabled us to choose a cutoff point for the test more accurately based on the scores of the healthy subjects. Furthermore, this was a small sample size, and because of this, caution should be used when interpreting the findings. Additionally, no formal sample size calculation was conducted, although methodological guidance for sample size determination in diagnostic accuracy studies is available in the literature [26]. Moreover, we have only tested stroke patients with right hemisphere damage, so we do not know how people with left hemisphere damage will react. In addition, in our study, all participants had a FAC score of 3 or higher. Individuals with lower FAC scores are able to perform the test in a seated position. We do not expect the seated variant to affect test outcomes; however, this cannot be stated with certainty. Another limitation that should be highlighted is that there is no gold standard for diagnosing “extrapersonal neglect.” The CBS comes closest, as it includes extrapersonal, personal, and peripersonal neglect. However, it gives no separate score for these individual regions. In future validity studies, we would recommend using the KF‐NAP to score the CBS, as its more specific scoring guidelines ensure a standardized test administration. Finally, to perform the test, you need a GRAIL system. To enhance the clinical feasibility of the FEPSNeT, the concept of the test could be rolled out on more affordable hardware.

## 5. Conclusions

Even though the FEPSNeT was considered to measure the construct visual neglect, the outcomes of the test were not significantly correlated with paper‐and‐pencil tests (SCT and LBT). Hence, it appears that the FEPSNeT detects neglect in a different region (extrapersonal space) than the paper‐and‐pencil tests (peripersonal space). Therefore, the FEPSNeT can be considered of additional value to paper‐and‐pencil tests to assess the multidimensional facets of neglect. However, more research is needed because only one group of stroke patients (with right hemisphere damage) has been tested, and because we did not test healthy subjects.

## Author Contributions

**Wieteke Sauter:** conceptualization, data curation, formal analysis, investigation, methodology, project administration, supervision, writing—original draft, writing—review & editing. **Peter van der Wurff:** conceptualization, methodology, supervision, writing – original draft, writing—review & editing. **Nikita van Schijndel:** conceptualization, investigation, writing—review & editing. **Pim Abbink:** conceptualization, investigation, writing—review & editing. **Noël Keijsers:** conceptualization, investigation, writing—review & editing. **Juha Hijmans:** conceptualization, writing—review & editing. **Kenneth Meijer:** conceptualization, writing—review & editing. **Anke van Bladel:** conceptualization, writing—review & editing. **Maarten R. Prins:** conceptualization, data curation, formal analysis, methodology, software, supervision, writing—original draft, writing—review & editing.

## Funding

No funding was received for this manuscript.

## Ethics Statement

The ethical standards of the Helsinki Declaration of 1975 and revised in 2014 were followed (General Assembly of the World Medical Association, 2014). On September 6, 2016, the medical ethical committee of UMCG (University Medical Center Groningen) determined that no medical ethical judgment was required for this study because data were collected during usual care (METc2016/306 UMCG).

## Consent

All subjects participated voluntarily in the study, and all subjects provided written informed consent prior to participating in this study.

## Conflicts of Interest

The authors declare no conflicts of interest.

## Data Availability

The data that support the findings of this study are available on request from the corresponding author. The data are not publicly available due to privacy or ethical restrictions.
